# Comparison of Wound Healing Efficiency Between Bacterial Cellulose Dry Membrane and Commercial Dressings

**DOI:** 10.3390/jfb16100366

**Published:** 2025-10-01

**Authors:** Wei-Wen Sung, Yu-Jing Zeng, Tsung-Ming Yeh, Yao-Yuan Chen, Min-Kung Hsu, Sung-Pin Tseng, Hsian-Yu Wang

**Affiliations:** 1 General Research Service Center, National Pingtung University of Science and Technology, Pingtung 912301, Taiwan; wwsung@mail.npust.edu.tw (W.-W.S.); yjz@mail.npust.edu.tw (Y.-J.Z.); ytm@mail.npust.edu.tw (T.-M.Y.); 2 Graduate Institute of Animal Vaccine Technology, National Pingtung University of Science and Technology, Pingtung 912301, Taiwan; s29947@gmail.com (Y.-Y.C.); afukada@gmail.com (M.-K.H.); 3 Department of Medical Laboratory Science and Biotechnology, College of Health Sciences, Kaohsiung Medical University, Kaohsiung 807378, Taiwan; 4 Center for Tropical Medicine and Infectious Disease Research, Kaohsiung Medical University, Kaohsiung 807378, Taiwan

**Keywords:** bacterial cellulose membrane, dressing, wound healing

## Abstract

The development of dressing materials mainly protects the wound, prevents infection, and assists in wound healing. Apart from the most common gauze on the market, different dressing materials can accelerate wound healing. Bacterial cellulose (BC) dressings have had many related studies and applications so far, and other natural or artificial compounds that are beneficial to tissue repair may also be added during the manufacturing process. This study compared the wound healing efficacies of BC dry membrane developed by our team, gauze, commercially available “Tegaderm^TM^ Hydrocolloid Dressing”, and “AQUACEL^®^ EXTRA Hydrofiber Dressing”. This study used rats as experimental animals and injured them by scalding. Moreover, *Staphylococcus aureus* was used to infect wounds to compare the effects on wound healing. We first used NIH-3T3 cells for an in vitro model to confirm that the BC membrane is not harmful to cells. In the animal experiment, wounds were created by scalding and then treated with different dressing materials and doses of *S. aureus*. After 10 days of treatment, the wound recovery in the BC membrane and AQUACEL^®^ groups was the most obvious, including angiogenesis in the dermal layer and regeneration of the epidermis layer. Especially without *S. aureus* infection, inflammatory markers such as cyclooxygenase-2 (COX-2) and inducible nitric oxide synthase (iNOS) expression levels were reduced to those of healthy tissue. In conclusion, we confirmed that the BC dry membrane can accelerate wound healing. In the future, it may provide high-efficiency and less expensive options in the dressing market.

## 1. Introduction

Skin wound healing is a highly complex process involving inflammatory response, cell proliferation, and differentiation. The inflammatory response includes an accumulation of neutrophils and macrophages and the release of growth factors to trigger vascular regeneration [1]. At the same time, fibroblasts and keratinocytes induce epithelialization by secreting connective-tissue matrix [2]. In addition, during the process of cell remodeling, type III collagen matures and transforms into type I collagen. Type I collagen is one of the main components of the extracellular matrix (ECM). It can give elasticity and tension to new tissue and prevent it from breaking due to excessive stretching [3]. Among the skin injuries, burns are more severe and have a higher risk of infection. Different dressings have been developed and produced to protect the wound and accelerate healing. Among the various kinds of dressing, gauze has been the most commonly used material for a long time due to its cheapness. However, because of its high stickiness to new tissue, especially when there are a lot of exudates, it is more difficult to replace. It can easily cause repeated injuries, which will slow down the healing speed of the wound. Therefore, dressings made from various materials have been developed recently to ease replacement, prevent infection, and accelerate healing.

In the current development of dressing materials, bacterial cellulose (BC) has attracted attention due to its high biocompatibility, hydrophilicity, and low cytotoxicity [4,5]. BC is the extracellular polysaccharide metabolite secreted from certain bacteria, which is polymerized from D-glucose or glucopyranose through the unbranched β-1,4 linkage [6], and the fiber diameter is usually several nanometers to form an extremely thin natural membrane. The polysaccharide chains form the subfibrils and further into microfibrils. The final ribbon or bundle morphology has a dense cross structure [2]. Apart from being used as a dressing material in past applications, it has also been widely used in biomedical research such as tissue engineering, drug delivery, neuromedicine, vascular transplantation, or the cartilage scaffolds field [7,8,9,10]. In research on wound healing, previous studies have found that wounds treated with BC showed high tissue and vascular regeneration, which is helpful for wound healing with highly porous and biodegradable characteristics [11,12,13]. Moreover, several previous studies showed that some compounds, such as TiO_2_, alginate, collagen, resveratrol, or antibacterial agents, could increase BC’s biocompatibility and chemical properties [14,15,16,17].

In the past development of BC, most of them were present in the form of a wet solution or sprayed to form a film on the wound. The advantage is that it can provide a moist environment for the wound, which is helpful for the healing process, such as cell regeneration, and can also reduce the pain [18,19]. However, they may also have disadvantages, such as easy loss. In this study, we developed a dry-form BC membrane, whose fiber diameter is 2~100 nm and the thickness is 0.05 mm. This nanometer-level membrane has high biocompatibility, biodegradability, vapor permeability, and a complete barrier between viruses and bacteria.

Moreover, the membrane will not cause rejection and can naturally stick to skin wounds. In addition, the BC dry membrane is translucent and can help track the patient’s wound recovery. Here, animal experiments would be used to compare the wound healing effects of this BC dry membrane, gauze, and commercially available higher-grade dressings, including Tegaderm^TM^ Hydrocolloid Dressing (3M, Saint Paul, MN, USA, hereinafter referred to as “Tegaderm”) and AQUACEL^®^ EXTRA Hydrofiber Dressing (ConvaTec Inc., Reading, UK, hereinafter referred to as “Aquacel”). Moreover, microorganism infection of wounds is a prevalent complication [20,21]. Open wounds, such as burns and scalds, can quickly provide a favorable environment for the growth of microorganisms and further cause pathogenic infection around the wound and more severe tissue damage [22]. In addition to delaying wound healing, it is more likely to worsen the wound and even increase mortality [23]. In this study, different doses of *Staphylococcus aureus* will be applied to the wound to explore the impact of the degree of wound inflammation on the effectiveness of the dressing.

## 2. Materials and Methods

### 2.1. Bacterial Cellulose Membrane Production

The BC membrane used in this study was produced by *Acetobacter xylinum*, which was cultured in the medium described previously for cellulose biosynthesis [24]. Briefly, bacterial nanocellulose is produced from *Acetobacter xylinum* at the air-liquid interface. After production, it is harvested, washed, and homogenized into a fiber slurry. Through controlled redeposition and a drying process at 80 °C, reconstructed membranes are formed. These membranes are then cut, sterilized using radiation treatment, and packaged as aseptic biomedical materials.

### 2.2. Scanning Electron Microscopy (SEM)

The surface 3D BC structure was examined under the field emission scanning electron microscope (FE-SEM Hitachi-4700, Hitachi Ltd., Tokyo, Japan). The sample was fastened on the copper base and then plated with platinum using a vacuum deposition machine. The SEM image was observed under an accelerating voltage of 10 kV at a working distance of 15.0 mm.

### 2.3. Cell Culture, BC Extraction, and Cytotoxic Assay

NIH-3T3 cells, which are the fibroblast isolated from NIH/Swiss mouse embryo, were purchased from Bioresource Collection and Research Center (Hsinchu, Taiwan) and were cultured in Dulbecco’s modified minimal essential medium (DMEM), including 10% fetal bovine serum (FBS), at 37 °C in a humidified incubator supplied with 5% CO_2_.

For BC extraction, the dry membrane was soaked in cell-cultured medium (6 cm^2^/mL) and stirred at 100 rpm for 24 ± 1 h according to ISO 10993-12:2012 standard procedure [25], which is the guideline for sample preparation and reference materials for biological evaluation of medical devices. This standard procedure includes “How to select test samples”, “How to select representative parts of medical devices”, “Test sample preparation and control group selection and preparation”, “Reference material selection and requirements”, and “Extraction solution preparation”. After extraction, the extract was diluted into different concentrations using a cultured medium for subsequent in vitro cytotoxic assay.

3 × 10^5^ cells/well of NIH-3T3 cells were seeded into the 6-well cultured plate for the cytotoxic qualitative experiment. After 24 h of incubation, the cultured medium was removed and replaced by different concentrations of BC membrane extract for experimental groups, cultured medium only for the negative control group, and medium containing Camptothecin (CPT) for the positive group [26], respectively. The cell morphology was observed under a light microscope after 24 and 48 h of treatment. The qualitative morphological grading of cytotoxicity was classified into grade 0 (none), 1 (slight), 2 (mild), 3 (moderate), and 4 (severe) according to ISO 10993-5:2009 standard [27], which is part of the biological evaluation standards for medical devices, focusing on in vitro cytotoxicity testing. It assesses the effects of materials or their leachable substances on cell viability and morphology through direct contact, indirect contact, or extract methods, serving as a preliminary safety screening.

In the quantitative assay, 1 × 10^5^ cells/well of NIH-3T3 cells were seeded into the 96-well culture plate. The cells were treated under conditions in a qualitative experiment. The cytotoxic assay was performed using CellTiter 96^®^ AQueous One Solution Cell Proliferation Assay (MTS) Kit (Promega, Madison, WI, USA) after 48 h of treatment, and the results were measured under OD_590_ using the Varioskan LUX multimode microplate reader (Thermo Fisher Scientific Inc., Waltham, MA, USA).

### 2.4. S. aureus-Containing Poloxamer 407 Hydrogel Preparation

The frozen *S. aureus* stock was purchased from Bioresource Collection and Research Center (Hsinchu, Taiwan) and recovered through culturing on the Lysogeny broth (LB) agar plate and subsequently in LB broth. Once the cell density was achieved, the whole *S. aureus* cells were centrifuged as pellets and resuspended as 1 × 10^7^ CFU/mL and 1 × 10^9^ CFU/mL in a cold 30% poloxamer 407 solution. As the temperature rises to room temperature, the *S. aureus* suspension would turn from liquid to gel form for subsequent application to the rat’s wounds.

### 2.5. Animal Experiment

Twelve six-week-old male Sprague Dawley (SD) rats were purchased from BioLASCO Taiwan Co., Ltd. (Taipei, Taiwan) and were adapted to the negative-pressure animal facility for one week. The animal experiment was approved by the Institutional Animal Care and Use Committee of National Pingtung University of Science and Technology (NPUST-112-070). The rats were fed under 25 ± 1 °C temperature, 55 ± 5% relative humidity, and a 12:12-hr light: dark cycle. The rats were divided into 12 groups for the experiment, and the dorsal hair was removed. Next, a 100 °C round metal rod was placed on each side of the hair-removed area on the rat’s back for 10 s to create a second-degree burn wound with a diameter of 2 cm [28]. The necrotic skin caused by the burn was removed the next day. The different dressings were subsequently applied onto the injured skin, which included sterile gauze (Group 1, 5, 9), dry BC membrane (Group 2, 6, 10), Aquacel (Group 3, 7, 11), and Tegaderm (Group 4, 8, 12). All dressings were individually packaged in sterile sheets and stored at room temperature before use. During the experiment, the packaging was opened with sterile scissors, and the dressings were cut into appropriate sizes before being applied directly to the wound. For both the BC and Tegaderm groups, the dressings were layered with gauze and secured in place with a bandage. In addition, low (1 × 10^7^ CFU, Group 5–8) and high dosages (1 × 10^9^ CFU, Group 9–12) of *S. aureus* were challenged and smeared between the injured skin and dressing. In the experiment, the gauze for groups 1, 5, and 9 was changed daily and moistened with normal saline to facilitate its removal. For the other groups, the dressing was changed when the exudate became excessive and the dressing fell off naturally. If it was still slightly sticky during the change, it was also moistened with normal saline. After 10 days from dressing treatment, the rats were sacrificed using CO_2_, and the skin of the wound area was harvested for further analysis.

### 2.6. Histological Analysis and Blood Vessel Count

The lesion skin was fixed with 10% formalin solution, embedded in paraffin, and subsequently cut into a 5-μm-thick section. The sections were subjected to hematoxylin and eosin (H&E) staining, and the results were finally observed under the light microscope. For counting blood vessels, five different fields of view within the wound area were randomly selected after magnifying the sample to 200× under a microscope. The number of blood vessels in each field of view was then counted, and subsequent statistical analysis was performed.

### 2.7. Protein Extraction and Western Blot

The rat skin was homogenized in T-PER^TM^ Tissue Protein Extraction Reagent (Thermo Fisher Scientific Inc., Waltham, MA, USA) containing the protease inhibitor cocktail using a sonicator and then centrifuged to remove tissue debris. The extracted proteins were quantified using Bio-rad Protein Assay Dye Reagent (Bio-Rad Laboratories, Hercules, CA, USA) according to the manufacturer’s instructions. Subsequently, the protein samples were resolved in the 10% acrylamide gel using SDS-PAGE and transferred onto Hybond^TM^-P Membrane (Amersham Biosciences, Amersham, UK). Following blocking with 5% non-fat milk in a phosphate-buffered system (PBS) containing 0.05% Tween-20 (PBS-T), the membranes were incubated with COX-2 (ABclonal, BioAb Co., Ltd., New Taipei, Taiwan), iNOS (Santa Cruz Biotechnology, Inc., Dallas, TX, USA), and β-actin (Cell Signaling Technology, Inc., Boston, MA, USA) primary antibodies overnight at 4 °C. After washing by PBS-T, the membranes were subsequently hybridized with Horseradish peroxidase (HRP)-conjugated goat anti-rabbit secondary antibody (Bio-Rad Laboratories) for 1 h at room temperature. After washing thoroughly and adding Western Lightning^TM^ *Plus*-ECL (Perkin Elmer Inc., Shelton, CT, USA), the results were visualized under ChemiD^TM^ XRS+ Imaging Systems (Bio-Rad Laboratories).

### 2.8. Statistical Analysis

The data from the quantitative cytotoxic assay were obtained from three independent experiments and are presented as the mean ± standard deviation. Statistical analysis was conducted using an unpaired *t*-test. Additionally, blood vessel counts were expressed as the mean ± standard deviation of the number of blood vessels observed in five visual fields for each group, with analysis performed using one-way Analysis of Variance (one-way ANOVA). A significant difference was determined at *p* < 0.05.

## 3. Results

### 3.1. BC Membrane Structure and Cytotoxic Assay

**Figure 1:**
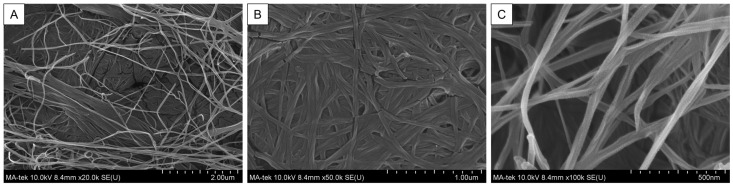
The surface morphology of the BC membrane was observed under SEM at (**A**) 20,000, (**B**) 50,000, and (**C**) 100,000× magnifications. The length indicated by the scale bar below each image means the total length of 10 grids.

The BC membrane structure and features were observed under the scanning electron microscope (SEM). The SEM images clearly show that the BC membrane has a highly interconnected fibrous network structure, with nanopores composed of randomly entangled cellulose to form a porous three-dimensional matrix. At low magnification, as shown in Figure 1A, the BC membrane presents an open, sponge-like structure. At the same time, in high-magnification images, the fibers are closely arranged, with a single fiber diameter of approximately 30–80 nm (Figure 1B,C). This unique nanoscale fiber morphology provides a large specific surface area and well-connected pores, which are beneficial for wound dressing applications, facilitating efficient liquid absorption, gas exchange, and promoting cell infiltration to support wound healing.

**Figure 2:**
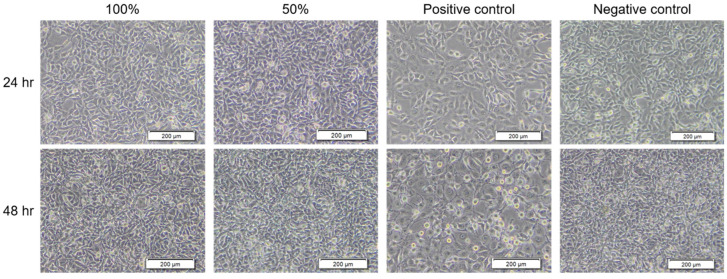
Microscopic observation for qualitative cytotoxic analysis of NIH-3T3 cells treated with different concentrations of BC extract, positive control (CPT), or negative control (medium only) for 24 and 48 h. The scale bar at the lower right corner of each photo = 200 μm.

**Figure 3:**
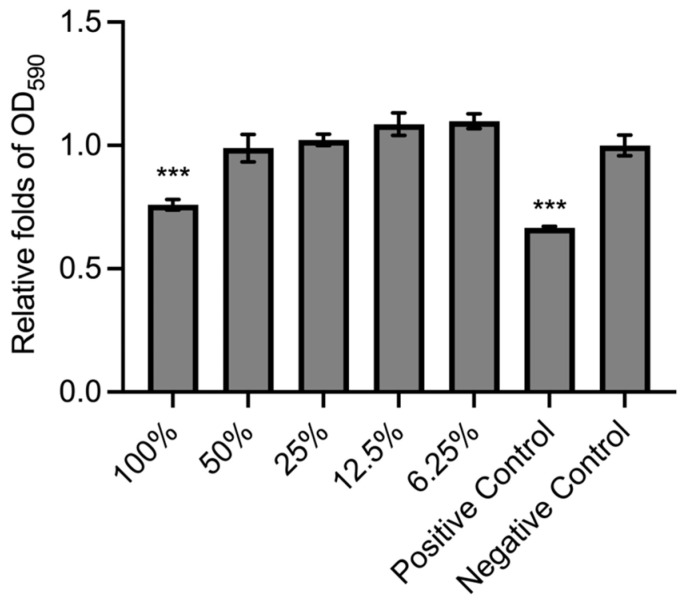
Quantitative cytotoxic assay of NIH-3T3 cells treated with different concentrations of BC extract, positive control (CPT), or negative control (medium only) for 48 h. The OD_590_ values were normalized by the mean value of the negative control group. All the data were performed under three independent experiments and shown as Mean ± SD. *** indicated *p* < 0.001 compared with the negative control group.

To verify that the BC membrane product has no harm to cell viability, the membrane extract was subjected to the treatment of NIH-3T3 cells following the ISO 10993-5:2009 standard. For the result of the qualitative experiment, as shown in Figure 2, the morphology of BC extract-treated cells was similar to that of the negative control. Also, the cytotoxicity grading revealed no damage to NIH-3T3 cells (Table 1). Moreover, in the quantitative experiment, the groups of 100% BC extract treatment and positive control showed a significant decrease compared to the negative control (Figure 3). However, the BC extract was identified as having no toxicity to cells because the survival rate of cells was higher than 70% (75.89 ± 2.12%) compared with the negative control (100 ± 4.21%) according to the “Reduction of cell viability by more than 30% is considered a cytotoxic effect” in ISO 10993-5:2009 standard. Meanwhile, under CPT treatment, the positive control had toxicity for NIH-3T3 cells (66.54 ± 0.65%). The results demonstrated that the BC membrane product has no cytotoxicity.

According to the “Qualitative morphological grading of cytotoxicity of extracts” in ISO 10993-5:2009 standard:

**Grade 0 (None):** Discrete intracytoplasmatic granules, no cell lysis, no reduction of cell growth.

**Grade 1 (Slight):** No more than 20% of the cells are round, loosely attached and without intracytoplasmatic granules, or show changes in morphology; occasional lysed cells are present; only slight growth inhibition observable.

**Grade 2 (Mild):** No more than 50% of the cells are round, devoid of intracytoplasmatic granules, no extensive cell lysis; not more than 50% growth inhibition observable.

**Grade 3 (Moderate):** No more than 70% of the cell layers contain rounded cells or are lysed; cell layers not completely destroyed, but more than 50% growth inhibition observable.

**Grade 4 (Severe):** Nearly complete or complete destruction of the cell layers.

### 3.2. Wound Healing Activity Between Different Treatments

**Figure 4:**
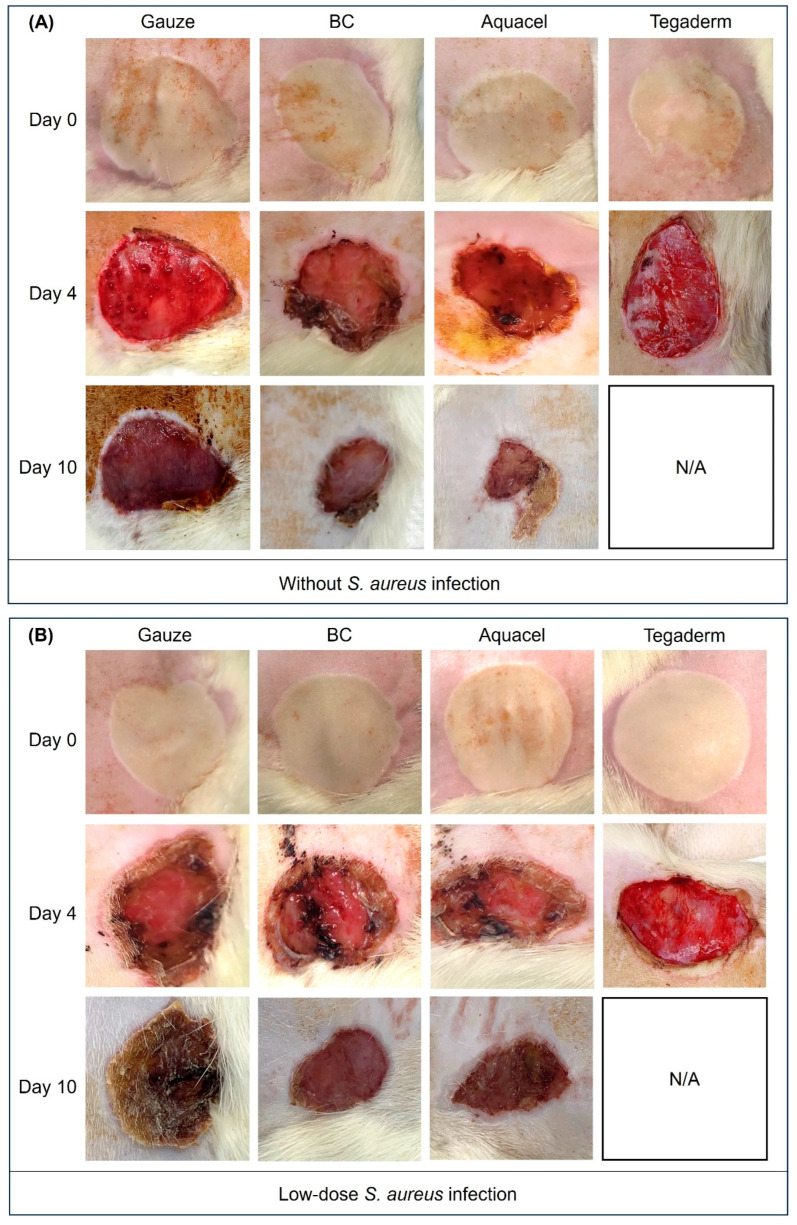

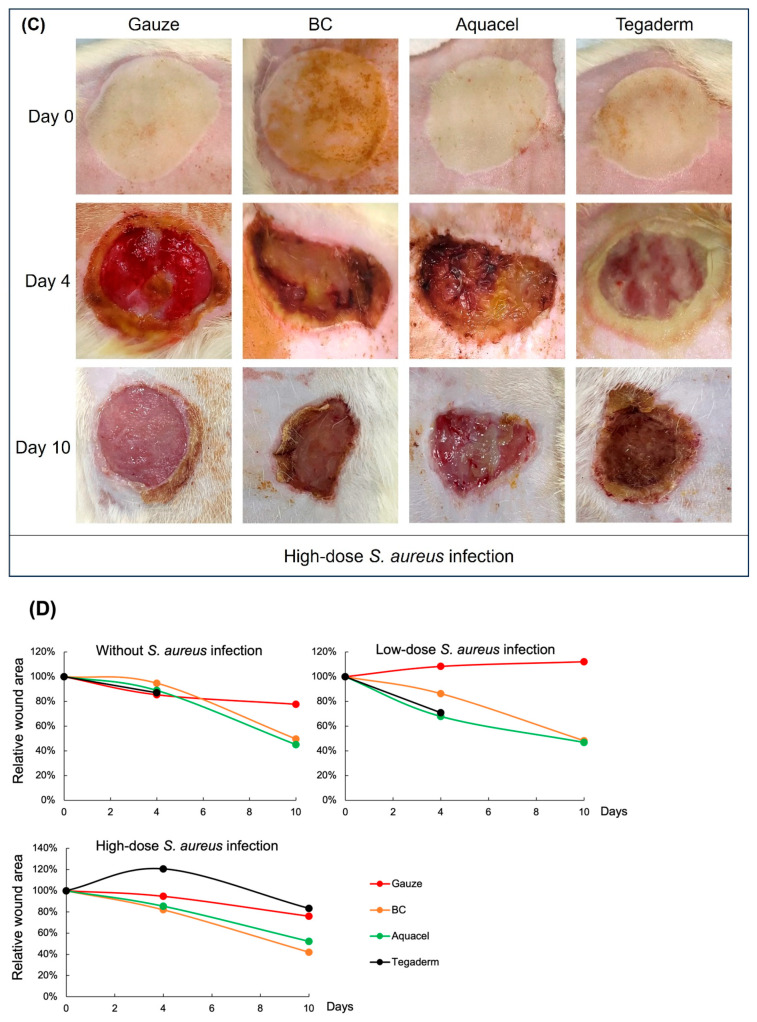
Photographs depicting wound healing conditions under various dressing treatments and different doses of *S. aureus* infection are shown as follows: (**A**) without infection, (**B**) low-dose infection, and (**C**) high-dose infection. In cases (**A**,**B**), the “N/A” indicates that the rats in the Tegaderm-treated group died on day 4, which is why there are no day 10 photos for these two groups. (**D**) The wound area ratios for days 4 and 10 were calculated using ImageJ software for each group, with the area on day 0 set as 100%.

The wound healing conditions were photographed after burn treatment and on day 4 and 10, as shown in Figure 4. However, due to excessive exudation during wound healing in the Tegaderm-treated group, the groups without *S. aureus* infection and the low-dose *S. aureus*-infected groups died on day 4, so there was a lack of photos of day 10 in these two groups in Figure 4A,B. On day 4 after burn treatment, the wounds of BC membrane and Aquacel treatments tended to dry and shrink without *S. aureus* infection (Figure 4A). In addition, in the BC-treated group, the dressing was found to have dried and formed a temporary scab-like state (Figure S1). However, the other two dressings still showed evident exudate. Moreover, the exudate was also present in all dressing treatments under low or high doses of *S. aureus* challenge (Figure 4B,C). After day 10 of treatment, the wound healing status treated by BC membrane and Aquacel was more evident than that of the other two, without and with a low dose of *S. aureus* infection. In the condition of a high dose of *S. aureus* infection, only the wound of the BC membrane-treated group was scabbed and dry on day 10. The other three groups, including the Aquacel-treated group, still presented few exudates (Figure 4C). Further analysis of the changes in wound area for each group showed that both BC and Aquacel dressings were more effective than the other dressings, regardless of the presence of *S. aureus* infection. By day 10, the wound area had reduced by approximately 50% (Figure 4D). The results revealed that the BC membrane and Aquacel treatments group could similarly facilitate wound closure compared with the other two dressings without *S. aureus* infection.

### 3.3. Comparison of Different Dressing Treatments by Histology Analysis

**Figure 5:**
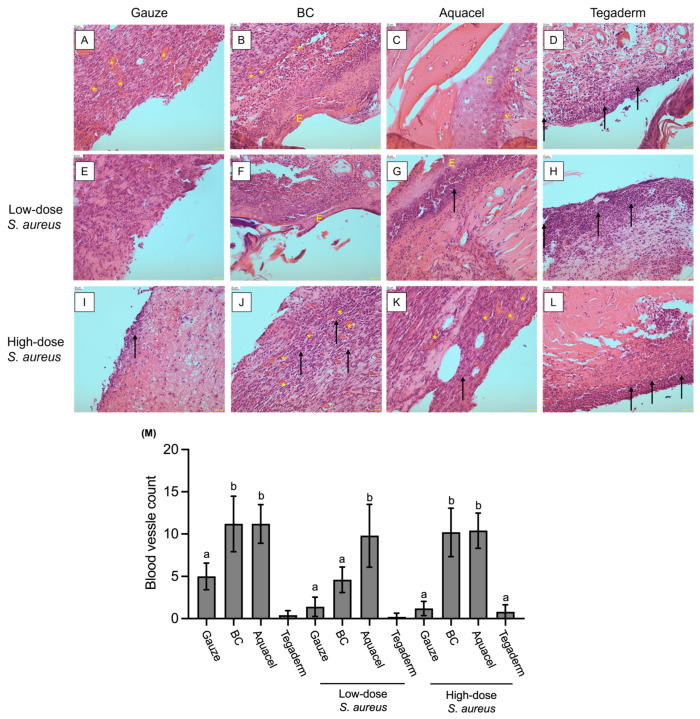
(**A**–**L**) The photomicrographs of H&E-stained injured skin under various dressings and *S. aureus* dose treatments, as shown in the diagram above and to the left at the endpoint of the experiment (day 4 for (**D**,**H**); day 10 for others). The black arrow and yellow asterisk indicate inflammatory infiltrate and blood vessels, respectively. The yellow “E” means epidermis layer. The scale bar at the upper left corner of each photo = 20 μm. (**M**) The results of blood vessel counts in the wound area are presented as Mean ± SD for five randomly selected fields. Different letters indicate significant differences (*p* < 0.05) among groups. It’s important to note that the results for the groups without and with low-dose *S. aureus* infection were obtained on day 4, while the results for the other groups were collected on day 10. As a result, statistical comparisons were not conducted between these groups.

The histology analysis was performed by H&E stain to examine the tissue recovery under different dressing treatments on day 4 without and low-dose *S. aureus* infected-Tegaderm groups, and on day 10 of the other 10 groups. The result images showed that the inflammatory infiltrate was observed in all treatments. However, there was no re-epithelialization or healing phenomenon under gauze-treated groups, regardless of the challenge with *S. aureus* (Figure 5A,E,I). Similar conditions were also found in Tegaderm-treated groups, although there were obvious inflammatory cells (Figure 5D,H,L). In addition, angiogenesis is also an essential process during wound healing [29], but there are fewer or no blood vessels under the treatments of gauze or Tegaderm.

For BC and Aquacel treatments, the re-epithelialization was found in the without and low-dose *S. aureus* challenge groups (Figure 5B,C,F,G), but not found in the high-dose *S. aureus* groups (Figure 5J,K). Additionally, as illustrated in Figure 5M, the number of blood vessels in the dermis of the wound regions indicated that both the BC and Aquacel groups exhibited significantly more angiogenesis than the other groups, regardless of *S. aureus* infection. The only exception was the low-dose *S. aureus*-infected BC group, which demonstrated fewer instances of angiogenesis; however, this was not statistically significantly different from the gauze and Tegaderm dressing groups. Despite this, the number of blood vessels in the low-dose *S. aureus*-infected BC group was still slightly higher.

Taken together, the results indicated that BC and commercial Aquacel dressing have a similar effect on improving wound healing due to the increasing angiogenesis, but infection with *S. aureus* would delay or inhibit the process because the epithelium layer was not found in the treatment of high-dose *S. aureus*. In the Tegaderm-treated group, the microscopic observation revealed intense inflammatory infiltration, but almost no angiogenesis was observed (Figure 5D,H,L). The gauze-treated groups also showed fewer blood vessels in the wound dermis layer and poor healing phenomenon.

### 3.4. Comparison of Inflammation Marker Levels Under Different Treatments

**Figure 6:**
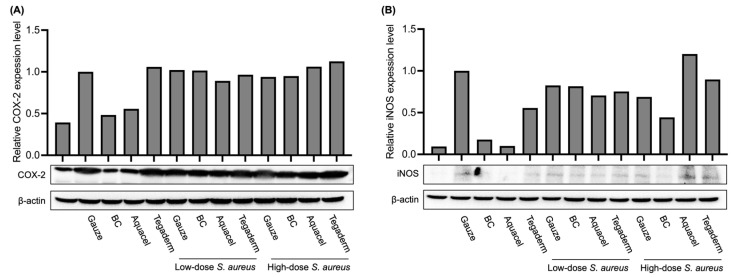
The expression levels of COX-2 (**A**) and iNOS (**B**) of the injured skin under different treatments at the endpoint of the animal experiment (day 4 for lanes 5 and 9; day 10 for others) by Western blot analysis. The quantitation was performed using Image J software (version 1.53a), and the intensity of the COX-2 or iNOS bands was divided by the β-actin intensity of the same sample. The value of the “gauze and no *S. aureus* infection” group (lane 2) was next used as the denominator for normalization.

In addition to histology observation, the expression levels of inflammation markers, such as cyclooxygenase-2 (COX-2) and inducible nitric oxide synthase (iNOS) in wound regions, were also examined by Western blot. As shown in Figure 6, in the skin that was not injured and not infected by *S. aureus*, COX-2 was expressed at a basic level (lane 1 in Figure 6A), while iNOS was expressed at a very low level (lane 1 in Figure 6B). In the case of burns, regardless of whether *S. aureus* was infected, the levels of both proteins increased in most groups, indicating obvious inflammation. It is worth noting that in the case of no *S. aureus* infection, the levels of COX-2 and iNOS in the groups treated with BC membrane and Aquacel were reduced to levels similar to those in the uninjured group (lanes 3 and 4 in Figure 6A,B). However, there was no effect of reducing the expression of these two proteins in the group treated with Tegaderm. The results of this part showed that if there is no severe infection, the BC membrane has a similar effect on reducing inflammation as Aquacel. After 10 days of treatment, its inflammation markers can be reduced to almost the same level as those of uninjured skin. However, there is no such effect in the gauze and Tegaderm groups.

## 4. Discussion

The primary purpose of this study was to compare the effects of several different dressing materials on wound healing. The type and composition of dressings play an important role in wound healing. In addition, whether there is microbial infection during the wound healing process will also affect the healing process [30]. Therefore, apart from comparing different dressing materials, this study also explores whether the wound is infected and the healing status. The BC membrane has been confirmed to have a structure similar to the extracellular matrix [31]. The results shown in Figure 1 also found that it has an irregular network structure, and the pores formed in the membrane are also conducive to the ingrowth of new fibroblasts [32]. These characteristics were also indicated as beneficial to the process of tissue repair. Moreover, BC membrane is classified as a bioactive dressing from biomaterials. The characteristics of the dressing are high biocompatibility, biodegradability, and low toxicity. The results of Figure 2 and Figure 3 also showed that this BC membrane is non-toxic to cells, thus confirming its safety for wound healing.

As mentioned, wound healing involves individual local or systemic regulation, including the interaction between growth factors, extracellular matrix, and various cytokines [33]. The use of dressings not only prevents infections caused by microorganisms, but their structure and composition may also play a role in accelerating healing. This study also compared the effects of gauze, Aquacel, and Tegaderm on the BC membrane. The results revealed that BC membrane and Aquacel had similar efficiency in wound healing without infection (Figure 4). On day 10, the wounds were significantly reduced and dried, while the gauze-treated group still had a small amount of exudate, and the wound size was still more significant than that of the other groups. Apart from comparing the effects of different dressing materials on wound healing under normal circumstances, this study also attempted to understand whether various dressing materials would change the healing status if the wound were infected by *S. aureus*. The results show that BC membrane and Aquacel were better than others, although the healing time was delayed, and the wounds were dry on day 10.

Generally, gauze is produced from cotton or non-woven fabric. Although gauze can absorb exudate, it is prone to sticking, causing regenerated cells or tissue to fall off when the gauze is torn off, delaying the healing process. Aquacel is composed of carboxymethylcellulose, and the characteristic is that it forms a gel after absorbing wound exudate and blocks the penetration of most pathogenic bacteria. Also, because of their colloid-forming properties, regenerated cells do not easily fall off when changing dressings [34,35]. Tegaderm is a semi-permeable film dressing made of polyurethane [19]. It is characterized by permeability and can prevent pathogenic infections [36]. However, this type of dressing has a limited ability to absorb exudate. Wounds with excessive exudate may cause infiltration of surrounding healthy tissue and increase the risk of infection [19]. Therefore, Tegaderm is more suitable for use in superficial or epithelializing wounds with less exudate.

On the other hand, inflammation within the wounds is also one of the indicators of healing. This study also used microscope observation of tissue sections to evaluate tissue repair and wound inflammation. The results showed that after 10 days of treatment, there were still varying degrees of inflammatory infiltration in the wounds of different groups. In the case of no or low-dose *S. aureus* infection, the reformation of the epidermis layer could be found under BC membrane and Aquacel treatment (Figure 5B,C,F,G). Under high-dose *S. aureus* infection, there is no epidermis layer formation; however, under BC membrane and Aquacel groups, more blood vessels could be found in the dermis layer than in others. This phenomenon reflected that the injured tissue was in a proliferation phase of tissue regeneration [37]. Moreover, in the absence of *S. aureus* infection, after 10 days of treatment in the BC membrane and Aquacel groups, interestingly, in addition to evident wound healing, the expression of inflammatory indicators such as COX-2 or iNOS was reduced to no significant difference from healthy tissue. However, in the case of *S. aureus* infection, the COX-2 and iNOS levels of the BC membrane and Aquacel groups were similar to those of other groups (Figure 6).

The wound-healing process includes redness, swelling, inflammation, blood vessel and cell proliferation, and tissue remodeling and maturation [38]. In this study, the gauze treatment groups were delayed for wound repair because the dressings were challenging to change. The images of tissue sections also demonstrated the small number of blood vessels after 10 days of treatment, which means that the phenomenon of cell proliferation was not apparent, and tissue necrosis may occur in the long term [4]. Additionally, due to the limited ability to absorb the exudate of Tegaderm, it was found that the dressing material often showed expansion and saturation by exudate during the experiment, causing infiltration of the surrounding skin and even slight ulceration. This may also be the reason why two groups of Tegaderm-treated rats died prematurely during the experiment. Therefore, it is not conducive to treating such deep or severely inflamed wounds.

The wound repair process of BC membrane and Aquacel groups is similar in terms of tissue sections and inflammation markers such as COX-2 and iNOS. In the absence of *S. aureus* infection, after 10 days of treatment with BC membrane or Aquacel, the expression levels of COX-2 and iNOS were lower than those in other groups and almost the same as healthy tissue. Previous research revealed that inflammation precedes angiogenesis and tissue remodeling once the injury is created [39,40]. Together with the results of tissue sections, it can be inferred that the wound tissue has recovered considerably. Even in the *S. aureus*-infected groups, the expression levels of COX-2 and iNOS are still high and similar to those of other dressing treatments. This means that the infection may delay the wound-healing process. However, it could also be found that angiogenesis or tissue remodeling was more noticeable compared with other dressing treatments. Both BC membrane and Aquacel can maintain moisture during wound healing, prevent water loss inside the wound, and prevent pathogens from penetrating and increasing the severity of infection. Unlike Aquacel, which formed a colloid after absorbing exudate, the BC membrane attached to the wound directly and dried to form an artificial scab if no excessive exudate was present. Subsequently, it healed and fell off naturally. It only needs to be moistened with normal saline if it needs to be removed. Previously, collagen, alginic acid, hyaluronic acid, etc., were added during the BC membrane production process to accelerate wound repair [41,42,43]. This study confirmed that the BC membrane itself has the function of accelerating the reconstruction of injured tissue.

## Figures and Tables

**Table 1:** Result of qualitative cytotoxic assay for NIH-3T3 cells.

Group	Length of Treatment Time (h)	Cell Morphology	Grading
BC extract	0	No cell lysis and reduction of cell growth	0
Negative control	0	No cell lysis and reduction of cell growth	0
Positive control	0	No cell lysis and reduction of cell growth	0
BC extract	24	No cell lysis and reduction of cell growth	0
Negative control	24	No cell lysis and reduction of cell growth	0
Positive control	24	Not more than 20% of the cells are round, only slight growth inhibition observable.	1
BC extract	48	No cell lysis and reduction of cell growth	0
Negative control	48	No cell lysis and reduction of cell growth	0
Positive control	48	Not more than 50% growth inhibition observable.	2

## Data Availability

The datasets in this study are available from the corresponding author on reasonable request.

## Supplementary Materials

The following supporting information can be downloaded at: https://www.mdpi.com/article/10.3390/jfb16100366/s1, Figure S1: Photographs of a wound treated with BC are shown for days 0 (A), 3 (B), and 6 (C).

## References

[1] Brassolatti P., Bossini P.S., Kido H.W., Derencio Oliveira M.C., Almeida-Lopes L., Zanardi L.M., Napolitano M.A., Retto da Silva de Avó L., Araújo-Moreira F.M., Parizotto N.A. (2018). Photobiomodulation and bacterial cellulose membrane in the treatment of third-degree burns in rats. J. Tissue Viability.

[2] Meng E., Chen C.L., Liu C.C., Liu C.C., Chang S.J., Cherng J.H., Wang H.H., Wu S.T. (2019). Bioapplications of bacterial cellulose polymers conjugated with Resveratrol for epithelial defect regeneration. Polymers.

[3] Plucknett D.L., Smith N.J. (1982). Agricultural research and Third World food production. Science.

[4] Khalid A., Ullah H., Ul-Islam M., Khan R., Khan S., Ahmad F., Khan T., Wahid F. (2017). Bacterial cellulose–TiO_2_ nanocomposites promote healing and tissue regeneration in burn mice model. RSC Adv..

[5] Volova T.G., Shumilova A.A., Nikolaeva E.D., Kirichenko A.K., Shishatskaya E.I. (2019). Biotechnological wound dressings based on bacterial cellulose and degradable copolymer P(3HB/4HB). Int. J. Biol. Macromol..

[6] Ross P., Mayer R., Benziman M. (1991). Cellulose biosynthesis and function in bacteria. Microbiol. Rev..

[7] Castro C., Zuluaga R., Putaux J.-L., Caro G., Mondragon I., Gañán P. (2011). Structural characterization of bacterial cellulose produced by *Gluconacetobacter swingsii* sp. from Colombian agroindustrial wastes. Carbohydr. Polym..

[8] Kowalska-Ludwicka K., Cala J., Grobelski B., Sygut D., Jesionek-Kupnicka D., Kolodziejczyk M., Bielecki S., Pasieka Z. (2013). Modified bacterial cellulose tubes for regeneration of damaged peripheral nerves. Arch. Med. Sci..

[9] Moniri M., Boroumand Moghaddam A., Azizi S., Abdul Rahim R., Bin Ariff A., Zuhainis Saad W., Navaderi M., Mohamad R. (2017). Production and status of bacterial cellulose in biomedical engineering. Nanomaterials.

[10] Novaes A.B., Novaes A.B. (1997). Soft tissue management for primary closure in guided bone regeneration: Surgical technique and case report. Int. J. Oral. Maxillofac. Implant..

[11] Sahana T.G., Rekha P.D. (2018). Biopolymers: Applications in wound healing and skin tissue engineering. Mol. Biol. Rep..

[12] Chevallay B., Herbage D. (2000). Collagen-based biomaterials as 3D scaffold for cell cultures: Applications for tissue engineering and gene therapy. Med. Biol. Eng. Comput..

[13] Horue M., Silva J.M., Berti I.R., Brandão L.R., Barud H.D.S., Castro G.R. (2023). Bacterial cellulose-based materials as dressings for wound healing. Pharmaceutics.

[14] Fu L., Zhang J., Yang G. (2013). Present status and applications of bacterial cellulose-based materials for skin tissue repair. Carbohydr. Polym..

[15] Lin S.-P., Loira Calvar I., Catchmark J.M., Liu J.-R., Demirci A., Cheng K.-C. (2013). Biosynthesis, production and applications of bacterial cellulose. Cellulose.

[16] Moraes P.R.F.d.S., Saska S., Barud H., Lima L.R.d., Martins V.d.C.A., Plepis A.M.d.G., Ribeiro S.J.L., Gaspar A.M.M. (2016). Bacterial cellulose/collagen hydrogel for wound healing. Mater. Res..

[17] Abazari M.F., Gholizadeh S., Karizi S.Z., Birgani N.H., Abazari D., Paknia S., Derakhshankhah H., Allahyari Z., Amini S.M., Hamidi M. (2021). Recent advances in cellulose-based structures as the wound-healing biomaterials: A clinically oriented review. Appl. Sci..

[18] Winter G.D. (1962). Formation of the scab and the rate of epithelization of superficial wounds in the skin of the young domestic pig. Nature.

[19] Dhivya S., Padma V.V., Santhini E. (2015). Wound dressings—A review. Biomedicine.

[20] Calum H., Moser C., Jensen P., Christophersen L., Maling D.S., van Gennip M., Bjarnsholt T., Hougen H.P., Givskov M., Jacobsen G.K. (2009). Thermal injury induces impaired function in polymorphonuclear neutrophil granulocytes and reduced control of burn wound infection. Clin. Exp. Immunol..

[21] Vivcharenko V., Trzaskowska M., Przekora A. (2023). Wound dressing modifications for accelerated healing of infected wounds. Int. J. Mol. Sci..

[22] Omar S., Asif A., Douha S., Joshua B. (2016). Antimicrobial dressings for improving wound healing. Wound Healing-New Insights Into Ancient Challenges.

[23] Woodford N., Livermore D.M. (2009). Infections caused by Gram-positive bacteria: A review of the global challenge. J. Infect..

[24] Forng E.R., Anderson S.M., Cannon R.E. (1989). Synthetic medium for Acetobacter xylinum that can be used for isolation of auxotrophic mutants and study of cellulose biosynthesis. Appl. Environ. Microbiol..

[25] (2012). Biological Evaluation of Medical Devices—Part 12: Sample Preparation and Reference Materials.

[26] Ryan A.J., Squires S., Strutt H.L., Johnson R.T. (1991). Camptothecin cytotoxicity in mammalian cells is associated with the induction of persistent double strand breaks in replicating DNA. Nucleic Acids Res..

[27] (2009). Biological Evaluation of Medical Devices—Part 5: Tests for In Vitro Cytotoxicity.

[28] Mirmohammadsadegh N., Shakoori M., Moghaddam H.N., Farhadi R., Shahverdi A.R., Amin M. (2022). Wound healing and anti-inflammatory effects of bacterial cellulose coated with Pistacia atlantica fruit oil. DARU J. Pharm. Sci..

[29] Dorsett-Martin W.A. (2004). Rat models of skin wound healing: A review. Wound Repair Regen..

[30] Gallant-Behm C.L., Yin H.Q., Liu S., Heggers J.P., Langford R.E., Olson M.E., Hart D.A., Burrell R.E. (2005). Comparison of in vitro disc diffusion and time kill-kinetic assays for the evaluation of antimicrobial wound dressing efficacy. Wound Repair Regen..

[31] Czaja W.K., Young D.J., Kawecki M., Brown R.M. (2007). The future prospects of microbial cellulose in biomedical applications. Biomacromolecules.

[32] O’Brien F.J., Harley B.A., Yannas I.V., Gibson L.J. (2005). The effect of pore size on cell adhesion in collagen-GAG scaffolds. Biomaterials.

[33] Guo S., Dipietro L.A. (2010). Factors affecting wound healing. J. Dent. Res..

[34] Walker M., Hobot J.A., Newman G.R., Bowler P.G. (2003). Scanning electron microscopic examination of bacterial immobilisation in a carboxymethyl cellulose (AQUACEL) and alginate dressings. Biomaterials.

[35] Waring M.J., Parsons D. (2001). Physico-chemical characterisation of carboxymethylated spun cellulose fibres. Biomaterials.

[36] Moshakis V., Fordyce M.J., Griffiths J.D., McKinna J.A. (1984). Tegadern versus gauze dressing in breast surgery. Br. J. Clin. Pract..

[37] Young A., McNaught C.-E. (2011). The physiology of wound healing. Surgery.

[38] Rivera A.E., Spencer J.M. (2007). Clinical aspects of full-thickness wound healing. Clin. Dermatol..

[39] Strecker-McGraw M.K., Jones T.R., Baer D.G. (2007). Soft tissue wounds and principles of healing. Emerg. Med. Clin. N. Am..

[40] Wang P.H., Huang B.S., Horng H.C., Yeh C.C., Chen Y.J. (2018). Wound healing. J. Chin. Med. Assoc..

[41] Zayed H.S., Saleh S., Omar A.E., Saleh A.K., Salama A., Tolba E. (2024). Development of collagen-chitosan dressing gel functionalized with propolis-zinc oxide nanoarchitectonics to accelerate wound healing. Int. J. Biol. Macromol..

[42] Zhang M., Zhang Q., Chen X., Jiang T., Song P., Wang B., Zhao X. (2022). Mussel-inspired nanocomposite hydrogel based on alginate and antimicrobial peptide for infected wound repair. Int. J. Biol. Macromol..

[43] Sadlik J., Kosińska E., Słota D., Niziołek K., Tomala A., Włodarczyk M., Piątek P., Skibiński J., Jampilek J., Sobczak-Kupiec A. (2024). Bioactive hydrogel based on collagen and Hyaluronic acid enriched with freeze-dried sheep placenta for wound healing support. Int. J. Mol. Sci..

