# Supplementary material for: Comparison of Wound Healing Efficiency Between Bacterial Cellulose Dry Membrane and Commercial Dressings
Source: J Funct Biomater. 2025 Oct 1;16(10):366. doi: 10.3390/jfb16100366 (PMC12565006; doi:10.3390/jfb16100366)
Supplement: Supplementary file 1 [file jfb-16-00366-s001.zip › jfb-3683998-supplementary.pdf]

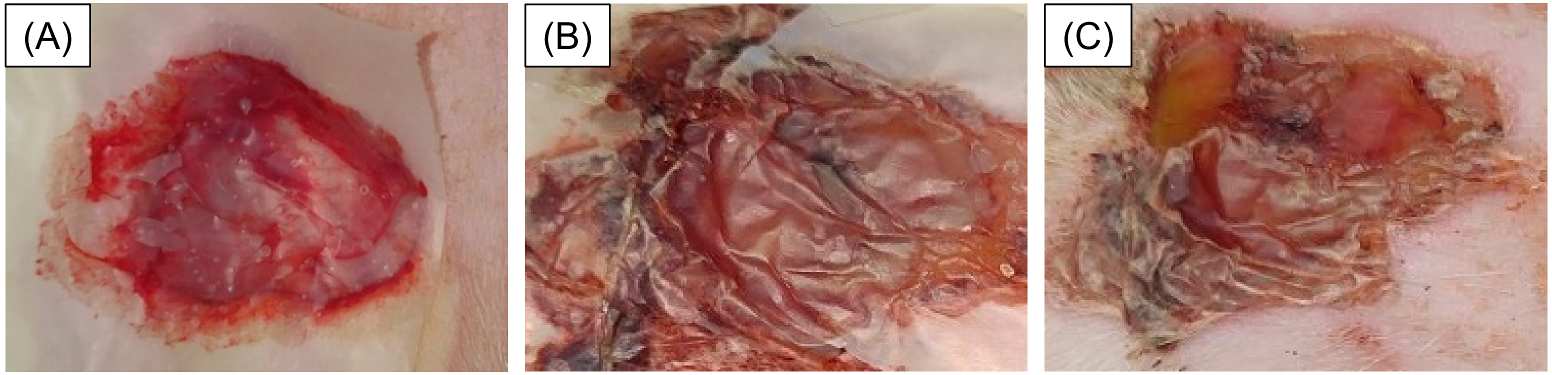

Figure S1. Photographs of a wound treated with BC are shown for days 0 (A), 3 (B), and 6 (C). By day 3, the BC dressing began to dry and formed a scab-like structure on the wound. In the absence of excessive exudate, this dry BC membrane will naturally peel off.
